# From Codons to Protein Structure: Evolutionary Constraints of Mitochondrial Proteins in Corvides

**DOI:** 10.3390/biology15141190

**Published:** 2026-07-19

**Authors:** Yingying Xiao, Mengsa Zhang, Mengtian Xi, Shiyun Han, Jianke Yang, Hui Peng, Wen Ge, Chenwei Dai, Lu Yang, Xianzhao Kan

**Affiliations:** 1 Anhui Provincial Key Laboratory of the Conservation and Exploitation of Biological Resources, College of Life Sciences, Anhui Normal University, Wuhu 241000, China; xiaoyingying@ahnu.edu.cn (Y.X.); zmshhh@ahnu.edu.cn (M.Z.); ximengtian@ahnu.edu.cn (M.X.); hansy@ahnu.edu.cn (S.H.); ajiankebc@wnmc.edu.cn (J.Y.); phui@wnmc.edu.cn (H.P.); gwen@whit.edu.cn (W.G.); 2 Anhui Rural Revitalization Collaborative Technology Service Center, Wuhu Institute of Technology, Wuhu 241003, China; 3 School of Basic Medical Sciences, Wannan Medical College, Wuhu 241002, China; 4 Teaching and Research Office of Evidence-Based Medicine, Wannan Medical College, Wuhu 241002, China; 5 Anhui Academy of Medical Sciences, Anhui Institute of Medicine, Hefei 230061, China; daicw@mail.ustc.edu.cn; 6 The Institute of Bioinformatics, College of Life Sciences, Anhui Normal University, Wuhu 241000, China

**Keywords:** mitochondrial proteins, protein structure, purifying selection, codon aversion, Corvides, functional constraints

## Abstract

Mitochondrial codon usage and selective pressures drive adaptive evolution and translation efficiency. This study provides insights into the evolutionary constraints shaping 13 core mitochondrial proteins in Corvides, based on 117 species, including 51 newly assembled genomes. We present evidence linking codon-level sequence architecture, protein structure, and selective pressures. These protein-coding genes (PCGs) are under strong purifying selection, with dN/dS ratios ranging from 0.00779 (*MT-CO1*) to 0.16214 (*MT-ATP8*). This conservation is reflected in the 3D model of MT-CO1, where conserved residues cluster within 12 transmembrane helices forming the core of its proton-pumping function. At the sequence level, we identify signatures of selection for translational efficiency, which are critical for accurate synthesis and folding. These signatures include a significant preference for “optimal” codons that perfectly match tRNA anticodons (*p* < 0.001). We also find lineage-specific features such as codon aversion motifs (CAMs). The *MT-ATP6* gene exhibits complete aversion of the CGA codon in *Oriolus chinensis* and of the ACT codon in *O. kundoo*. These sequence-level features resolve the deep phylogenetic relationships within the group, demonstrating the effectiveness of our multi-layered analytical framework. Overall, our results link codon-level sequence architecture with protein structural constraints, functional evolutionary signals, and mitochondrial genome evolution in Corvides.

## Simple Summary

Mitochondria are the powerhouses of living cells, but how their genes evolved in birds remains poorly understood. In this work, we studied genetic changes in 117 species of crows and related songbirds, aiming to uncover the evolutionary rules shaping the energy-producing proteins and their genetic codes. Our findings show that evolution strictly preserves the 3D shapes of these proteins, especially their core working regions, to ensure survival. We also discovered hidden rules in their genetic instructions: genes strongly prefer specific “words” for fast and accurate protein production, while completely avoiding others. This research decodes these unique genetic rules, helping us better understand bird evolution and providing a framework for future animal biodiversity studies.

## 1. Introduction

Mitochondria, the powerhouses of the eukaryotic cell, are distinctive organelles that possess their own genetic material and an independent system for transcription and translation [1,2,3,4]. Their function in cell proliferation, cell death, and energy metabolism depends on a compact set of co-evolved biological macromolecules encoded by the mitochondrial genome (mitogenome). These components, which include the 13 core mitochondrial proteins that form the core catalytic subunits of the oxidative phosphorylation machinery, alongside the 22 tRNAs and 2 rRNAs required for their synthesis, are essential for cellular survival [5]. Consequently, the structures, functions, and interactions of these proteins and RNAs are maintained under strong selective pressure [6]. A central goal in molecular biology is therefore to understand the evolutionary principles that shape their architecture and functional constraints [7].

The avian Corvides, a highly diverse group with nearly 800 species [8,9,10,11], provides a powerful model system for investigating the evolutionary dynamics of these components. Its global distribution and complex evolutionary history provide a rich comparative context to study how a conserved set of orthologous genes adapts and evolves across a wide phylogenetic landscape. Despite their importance, the structural and functional properties of the complete set of Corvides mitochondrial protein-coding genes (PCGs) and their protein products have yet to be fully investigated.

A critical but under-explored aspect of sequence-level architecture is codon usage bias (CUB), the unequal frequency of synonymous codons. This bias can directly influence the efficiency and accuracy of protein synthesis, and thus the final structure and function of the synthesized protein [12,13,14,15,16,17,18]. The efficiency of this process is influenced by interaction between mRNA codons and their corresponding tRNA anticodons. Specifically, codons recognized by abundant (“optimal”) codons are generally associated with enhanced translation efficiency and accuracy [19,20,21,22]. Codon aversion refers to the complete absence of one or more synonymous codons from a coding sequence. It is thought to result from changes in tRNA gene content together with selection on translational efficiency and accuracy. Because these patterns tend to be conserved within lineages while differing among taxa, codons provide a useful evolutionary signature for phylogenetic inference [23,24,25]. While studied in some plant and animal groups [26,27,28,29,30,31,32,33], how these architectural patterns vary across a large set of related PCGs and what forces drive them in a major vertebrate radiation remain poorly understood. Owing to their diverse ecology and rapid adaptive radiation, Corvides provide an excellent system for investigating sequence-level evolution. Codon usage patterns also complement analyses of protein-level selective pressures by distinguishing evolutionary forces acting on translational efficiency from those maintaining protein structure and function.

Selective pressures on mitochondrial PCGs were therefore evaluated by calculating the ratio of non-synonymous to synonymous substitution rates (dN/dS). This ratio provides an estimate of the strength and direction of selection acting on the amino acid sequence of a protein, which in turn determines its three-dimensional structure and function [34,35,36,37]. However, to fully understand these constraints, it is essential to visualize their physical manifestation. Three-dimensional structural modeling, coupled with conservation analysis, allows us to map evolutionary pressures directly onto the protein’s architecture. This reveals which domains, such as the catalytic core versus exposed loops, are most critical for function [38,39,40,41]. Previous studies in other avian groups have shown that these mitochondrial protein-coding genes are generally under strong purifying selection, but the specific rates vary significantly among them, from the highly conserved MT-CO1 protein to the fast-evolving MT-ATP8 protein [42,43,44]. Quantifying these selective pressures is particularly important for the Corvides, and is essential for understanding the functional constraints governing the core respiratory machinery in this major avian lineage.

In this study, we investigate the evolution of mitochondrial PCGs and their encoded proteins in Corvides. By assembling and annotating 51 new mitogenomes to generate a dataset of 117 species, we aim to: (1) characterize the compositional features of the 13 mitochondrial PCGs and their encoded proteins; (2) examine codon usage patterns and identify codon aversion motifs as potential evolutionary signatures; and (3) evaluate selective pressures and functional constraints. Together, these analyses provide insights into the evolution of mitochondrial PCGs and their utility for resolving the phylogeny of Corvides.

## 2. Materials and Methods

### 2.1. Data Collection, Assembly, and Annotation

To construct a comprehensive dataset of mitochondrial genes for the Corvides, raw genomic sequencing data were downloaded from the SRA database on 15 August 2025. We assembled the mitogenomes using GetOrganelle v1.7.3 [45], with *Acridotheres cristatellus* (NC_015613) serving as a reference. Following assembly, the newly generated circular mitogenomes were annotated using the GeSeq online tool [46]. To ensure accuracy and correct potential errors from the automated annotation process, we manually curated the annotations using BioEdit v7.2.6 [47]. Through this process, we obtained 51 newly assembled and annotated complete mitogenomes, and information on the contained protein-coding and RNA genes has been deposited into GenBank. Nucleotide sequence data are available in the GenBank databases under the accession numbers TPA: BK072017-BK072067 (Table S1).

To further expand the dataset, we retrieved 66 publicly available mitogenomes from the NCBI database. Our final dataset, representing the Corvides, comprises 117 mitogenomes from 22 families and 53 genera. The gene nomenclature follows the standards proposed by the HUGO Gene Nomenclature Committee [48].

### 2.2. Structural Modeling and Conservation Analysis

To investigate the physical basis of the observed functional constraints, we generated three-dimensional (3D) models for the 13 mitochondrial proteins. The amino acid sequences for each protein were used as input for structure prediction. We selected the most conserved protein (MT-CO1) and a representative fast-evolving protein (MT-ND2) for detailed comparative analysis. While MT-ATP8 exhibits the highest evolutionary rate, its small size and simple structure are not ideal for informative comparative structural analysis. Therefore, MT-ND2, as a rapidly evolving protein with a significantly larger and more complex structure, was selected to provide a more meaningful contrast.

The 3D structures were predicted using the ColabFold pipeline, which implements the AlphaFold2 algorithm [49,50]. For each protein, the top-ranked model based on the predicted local distance difference test (pLDDT) score was selected [51]. To visualize the functional constraints on the architecture of proteins, we calculated per-site conservation scores using the ConSurf server [52,53]. For this analysis, the predicted 3D structure of the protein and a multiple sequence alignment of the corresponding sequences from all 117 species were used as input. The resulting structures, colored by conservation scores, were then visualized and rendered using ChimeraX 1.10.1 [54].

### 2.3. Analysis of Sequence Architecture and Functional Constraints

#### 2.3.1. Codon Usage Patterns and Translational Selection

To investigate sequence-level architectural features that influence the translational efficiency and accuracy of protein synthesis, we first removed both the complete termination codons (AGG, AGA, TAA, and TAG) and the incomplete stop codons (T-). Subsequently, we calculated the effective number of codons (ENC), relative synonymous codon usage (RSCU), and the GC content of synonymous codons at the 3rd position (GC3s) using CodonW v1.4.4 [55].

The ENC is a key index for assessing codon usage bias, with its scale spanning from 20 (maximal bias) to 61 (uniform usage). Consequently, lower values reflect a stronger CUB, whereas higher scores are associated with more uniform codon usage [17,56]. Relative Synonymous Codon Usage (RSCU) measures the bias by comparing the observed occurrence of a particular codon to its predicted frequency, where a value > 1 suggests a preferred codon and a value < 1 indicates a negative bias [17,57]. The RSCU values were then used to generate a heatmap using TBtools 1.098 [58].

To investigate the factors shaping these codon usage patterns, we generated three diagnostic plots. The PR2-bias plot evaluated the effect of mutation versus selection by graphing AT-bias, calculated as A3/(A3 + T3), against GC-bias, calculated as G3/(G3 + C3), where deviation from the center (0.5, 0.5) reflects a violation of parity rule 2, indicating unequal utilization of complementary nucleotides [33,59,60]. The ENC-GC3s plot further distinguished between mutation pressure (points near or on the standard curve) and selection (points below) [56,61]. Finally, a neutrality plot correlating GC12 with GC3 was used to quantify these influences. A regression coefficient near 1 indicates that CUB is chiefly influenced by mutation, while a coefficient close to 0 suggests that CUB is primarily shaped by selection pressure for translational efficiency [33,61]. We also analyzed Codon Aversion Motifs (CAMs), for which codons with an RSCU value of 0 were defined as averted [62].

Furthermore, to directly test for a mechanism of translational selection acting on the biosynthesis of these proteins, we tested for co-evolution between codons and their corresponding tRNA anticodons. Based on mitochondrial wobble pairing rules, codons were classified into two groups: “optimal” codons, which form a perfect Watson–Crick pair with the tRNA anticodon, and “non-optimal” codons, which rely on wobble pairing [63]. Given the high conservation of tRNA anticodons among closely related avian species, the tRNA sequences from a representative species within the dataset (*Corvus corone*) were used to define the reference anticodons for this classification. We then compared the mean RSCU values between these two groups using a Mann–Whitney U test to determine if translational selection has favored the use of more efficiently translated codons. The distribution of RSCU values for each group was visualized using a box plot generated in R v4.4.1.

#### 2.3.2. Evolutionary Rates and Purifying Selection

To quantify evolutionary constraints on core mitochondrial protein, we calculated the rates of non-synonymous substitutions (dN), synonymous substitutions (dS), and their ratio (dN/dS) using the M0 model in PAML v4.9 [64]. This model was selected to estimate the overall evolutionary constraints across the 13 PCGs. The dN/dS ratio is a key indicator of selective pressure, where a value >1 indicates positive selection, =1 indicates neutral selection, and <1 suggests purifying selection [64,65,66,67].

### 2.4. Phylogenetic Validation of Phylogenomic Signals

To validate the strength and phylogenetic signal of the structural and functional signals derived from these PCGs, we constructed a sequence matrix for our phylogenetic analyses using PCGs from the 117 complete mitogenomes. We selected *Acanthisitta chloris* (NC_051004) and *Menura novaehollandiae* (NC_007883) as outgroups, and phylogenetic relationships were inferred through ML and BI approaches, respectively.

For the ML reconstruction, we performed the analysis in RAxML 8.2.12 [68], employing the GTRCAT model with 50 independent runs, and 1000 bootstrap replicates. The “autoMRE” criterion served to assess convergence. Regarding BI, we selected the most appropriate substitution model per gene locus using ModelTest-NG 0.1.6 [69] (Table S2). Then, MrBayes 3.2.7a [70] was employed for two parallel runs of four Markov chains each, extending for 5 million generations and sampling at intervals of 1000 generations. The final step involved using Tracer 1.7.1 [71] to check the effective sample sizes (ESS) of the merged runs for convergence.

## 3. Results

### 3.1. Functional Constraints and Structural Basis of the Core Mitochondrial Proteins

#### 3.1.1. Strong Purifying Selection Reveals Functional Importance

To quantify the functional constraints acting on the 13 core mitochondrial proteins of the Corvides, we first assessed the overall genetic variation in the genes that encode them. The percentage of variable sites (PV) differed substantially among the genes, with *MT-ATP8* being the most variable (PV = 69.70%) and *MT-CO1* being the most conserved (PV = 40.91%) (Table 1).

To disentangle the signature of functional constraint from the background of neutral variation, we analyzed dN and dS substitution rates. The high underlying genetic variation is reflected in the high and widely varied dS values (25.72–72.28). In striking contrast, dN values remained exceptionally low (0.3132–4.1706). Consequently, the dN/dS ratios of the 13 PCGs were all significantly below 1, ranging from 0.00779 for the highly conserved *MT-CO1* to 0.16214 for the fast-evolving *MT-ATP8* (Table 1).

It is noteworthy that while the overall evolutionary pattern is highly consistent (e.g., the *MT-CO* gene family is always the most conserved), the specific ranking of genes differs slightly when comparing metrics of overall variation (such as PV and π) against metrics of selective pressure (dN/dS). This divergence in ranking illustrates that the forces governing sequence-level mutation are distinct from the selective pressures that conserve protein function.

Ultimately, the consistently low dN/dS ratios provide strong evidence that these essential components of the respiratory chain have evolved under potent purifying selection.

#### 3.1.2. 3D Structures Visualize Functional Constraints

To understand the functional constraints from a physical perspective, we generated three-dimensional models for the most conserved protein (MT-CO1) and a representative fast-evolving protein (MT-ND2).

**Figure 1:**
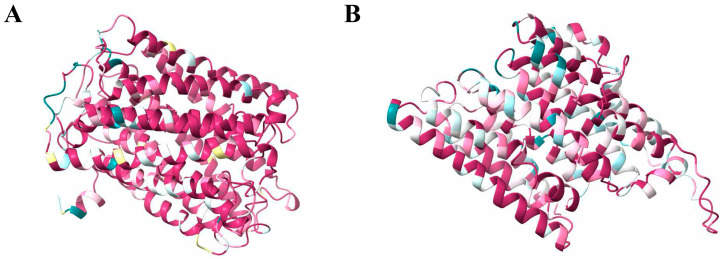
Comparison of surface conservation between two mitochondrial proteins. Three-dimensional models of (**A**) MT-CO1 and (**B**) MT-ND2 are shown with surfaces colored according to amino acid conservation levels. Deep magenta indicates highly conserved sites, whereas blue and white indicate highly variable sites, and yellow indicates residues with insufficient data. The surface of MT-CO1 in (**A**) appears largely conserved, while MT-ND2 in (**B**) exhibits a heterogeneous pattern of conserved and variable regions.

The highly conserved MT-CO1 protein serves as a clear example of stringent functional constraints. As shown in Figure 1A, its entire surface is dominated by a deep magenta color (conservation score of 9), indicating that its amino acid sequence has been subject to intense purifying selection throughout its evolution. These highly conserved residues are clustered within its 12 transmembrane helices, the core of its essential proton-pumping function. In stark contrast, the fast-evolving MT-ND2 protein displays a “mosaic” pattern of conservation (Figure 1B), featuring both conserved core regions and large, variable regions on its surface and in loop regions. This structural analysis visually demonstrates how functional constraints physically shape proteins.

### 3.2. Sequence-Level Architectural Features Influencing Protein Synthesis

#### 3.2.1. Codon Usage Patterns Reflect Selection for Translational Efficiency

To investigate the sequence-level architecture of the PCGs in the Corvides, we analyzed their CUB. The mean ENC values per gene ranged from 35.4 ± 4.75 (for *MT-ND4L*) to 43.62 ± 2.2 (for *MT-ND2*), indicating that the overall CUB was relatively weak across all 13 PCGs (Table S3).

**Figure 2:**
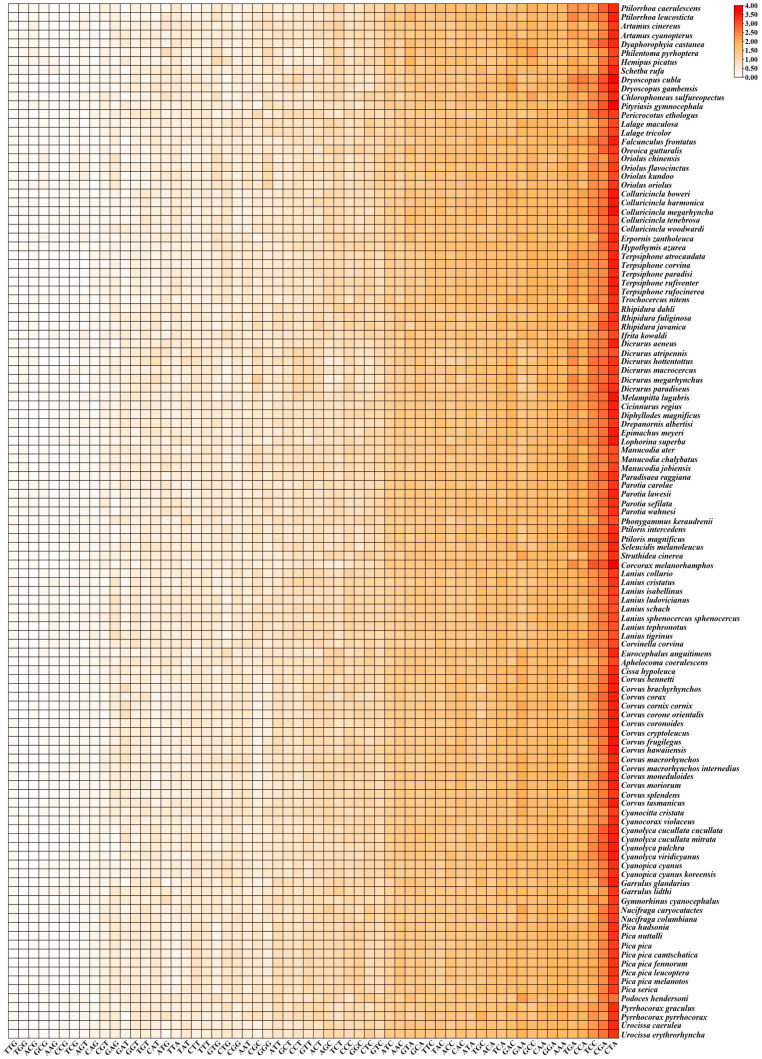
Heatmap of Relative Synonymous Codon Usage (RSCU) for the overall mitochondrial protein-coding genes (PCGs) of Corvides species. The color gradient reflects the frequency of codon usage, with warmer colors indicating a higher preference for specific codons.

Despite the weak overall bias, analysis of RSCU across the 13 PCGs revealed distinct preferences that act as architectural signatures (Figure 2 and Table S4). Consistent with other avian studies [72,73,74], codons ending in A or C were generally preferred (Table S5). For example, the codon with the highest RSCU value (3.33) was CTA, which encodes Leucine, the most frequently used amino acid. This alignment of codon preference with amino acid frequency suggests selection for translational efficiency of the most required protein components. A notable exception was the *MT-ND6* gene, which showed a distinct preference for codons ending with G or T.

**Figure 3:**
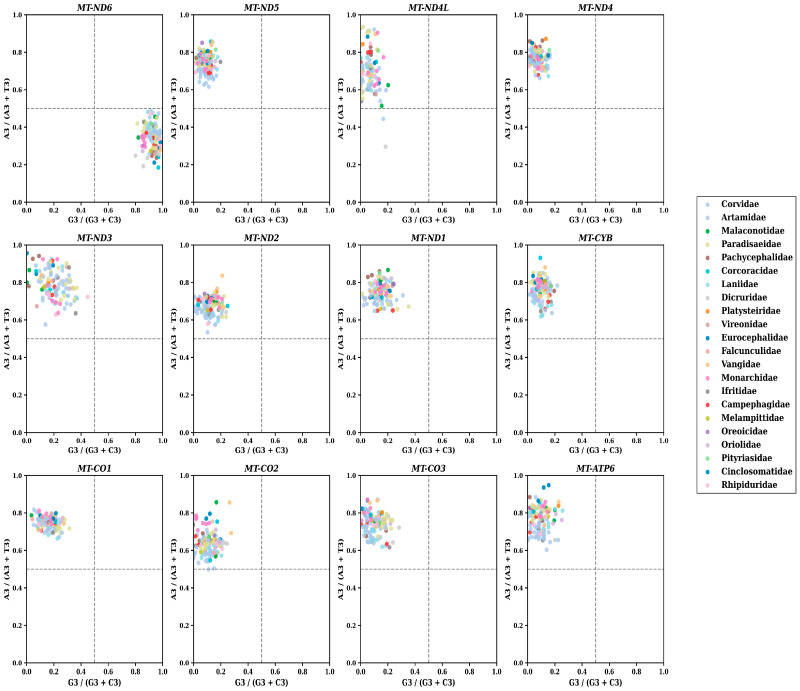
Parity Rule 2 (PR2) bias plots of mitochondrial genes among Corvides species. This plot evaluates the effects of mutation versus selection by illustrating the unequal utilization of complementary nucleotides. The *x*-axis represents GC-bias [G3/(G3 + C3)], and the *y*-axis represents AT-bias [A3/(A3 + T3)]. The dashed lines intersecting at (0.5, 0.5) represent the center point where A = T and G = C, indicating no bias. Different colors represent different families within Corvides, as indicated in the figure key.

**Figure 4:**
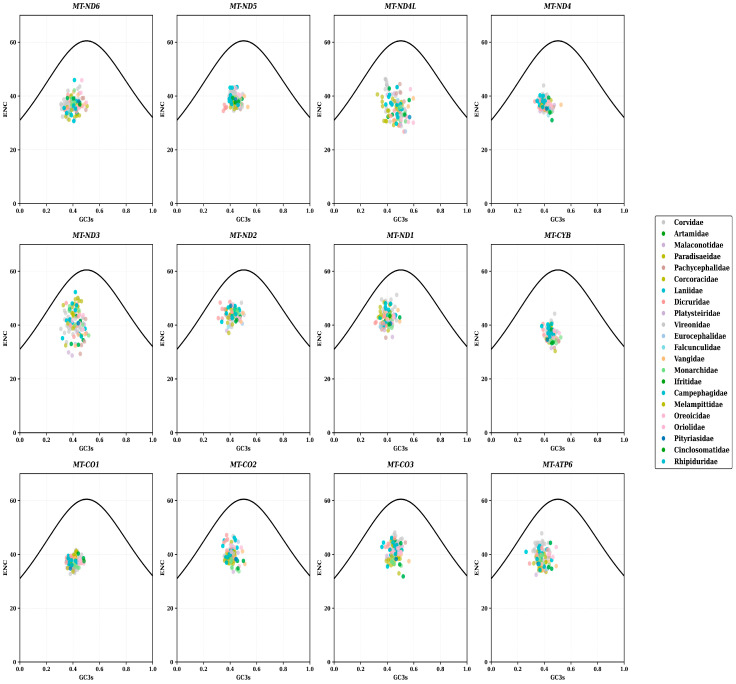
The comparison of the Effective Number of Codons (ENC) versus the GC content of synonymous codons at the 3rd position (GC3s) curve for the protein-coding genes (PCGs) in the mitogenomes of Corvides species. This plot is used to determine the primary factors shaping codon usage bias, where data points located considerably below the expected standard curve indicate that natural selection outweighs mutational pressure. Different colors represent different families within Corvides, as indicated in the figure key.

To elucidate the mechanisms responsible for these architectural patterns, a series of diagnostic plots indicated that natural selection, rather than mutational pressure, is the principal force shaping codon usage. The PR2 plot showed that most PCGs (except *MT-ND6*) are biased towards A and C at the third codon position, whereas *MT-ND6* is biased towards G and T (Figure 3). Furthermore, the ENC-GC3s analysis showed all data points located considerably below the expected curve (Figure 4), and neutral plots revealed exceptionally low slope values (ranging from 0.018 for *MT-CO1* to 0.139 for *MT-ND5*) (Figure S1), all of which suggest that selection outweighs mutational pressure.

#### 3.2.2. Preferential Usage of “Optimal” Codons

**Figure 5:**
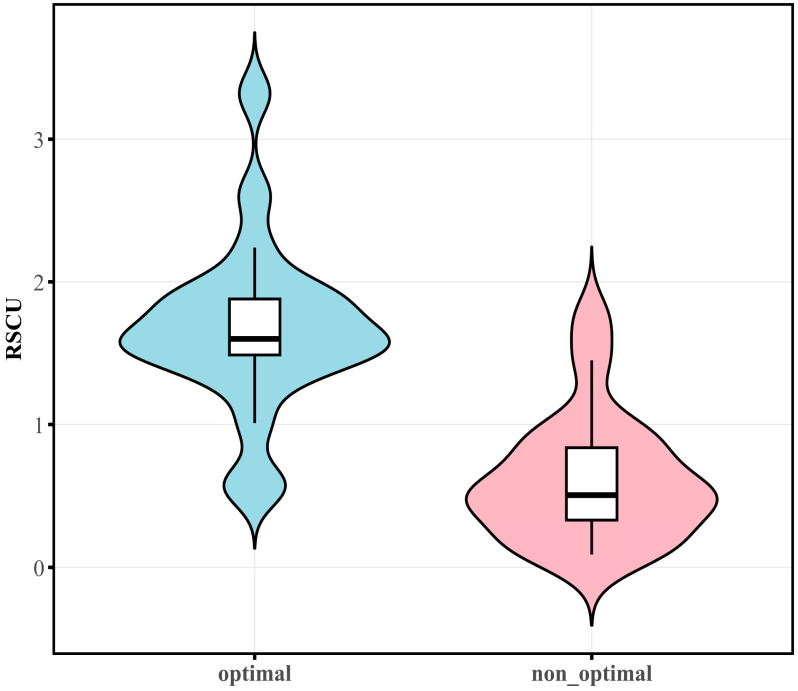
Distribution of RSCU for optimal and non-optimal codons across 13 PCGs. The median RSCU for the optimal group is significantly higher than that of the non-optimal group (Mann–Whitney U test, *p* < 0.001). The central box plot shows the median, interquartile range, and data whiskers.

To directly investigate the role of translational selection, we compared the usage of “optimal” codons (those forming perfect Watson–Crick pairs with the tRNA anticodon) against “non-optimal” codons. The analysis revealed that optimal codons are used significantly more frequently than non-optimal codons across the 13 PCGs (Mann–Whitney U test, *p* < 0.001). The median RSCU for the optimal group (median = 1.6, interquartile range [IQR] = 0.392) was substantially higher than that of the non-optimal group (median = 0.505, IQR = 0.5057) (Figure 5). This provides direct evidence that seletion for translational efficiency has played a key role in shaping these architectural features.

#### 3.2.3. Codon Aversion Motifs (CAMs) as Lineage-Specific Signatures

**Figure 6:**
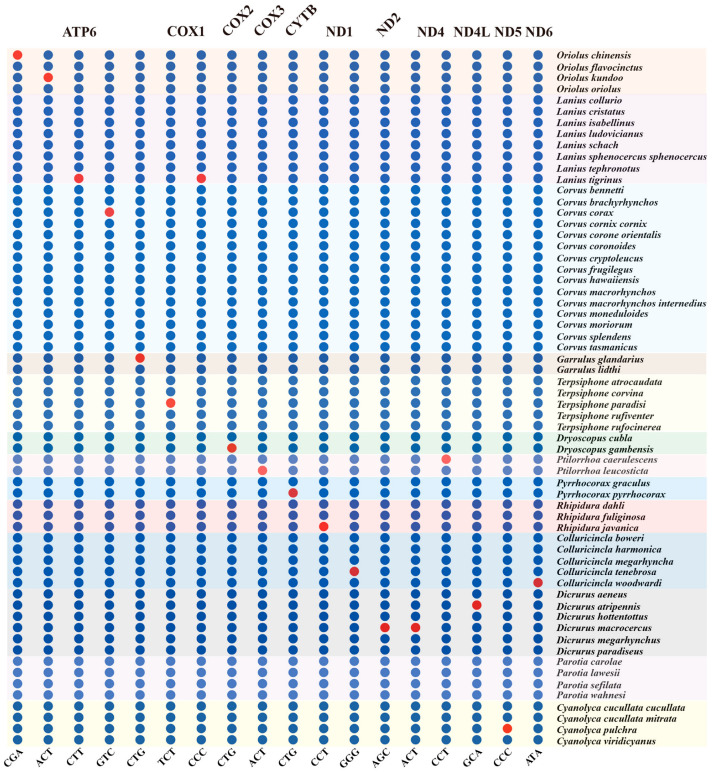
Distribution of CAMs across Corvides. Red circles represent complete codon aversion, while blue circles indicate presence. The different background colors indicate different genera.

A key example of these selected architectural signatures is CAM, the complete avoidance of specific codons. These CAMs serve as lineage-specific signatures with diagnostic potential. For instance, the gene encoding the MT-ATP6 protein exhibits unique CAMs within specific species, including the complete aversion of the CGA in *Oriolus chinensis*, ACT in *Oriolus kundoo*, CTT in *Lanius tigrinus*, GTC in *Corvus corax*, and CTG in *Garrulus glandarius* (Figure 6). These findings suggest that lineage-specific constraints may be acting on the translational machinery, further highlighting the tight co-evolution between the genetic code and the synthesis of these proteins.

### 3.3. Basic Compositional Properties of the PCGs and Their Protein Products

**Figure 7:**
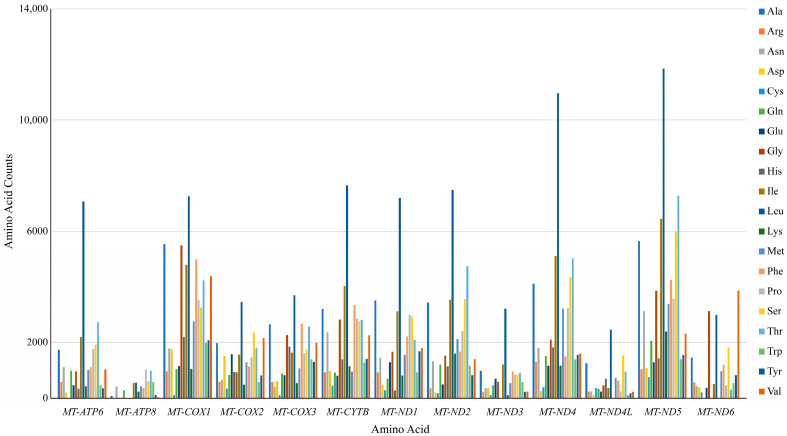
Amino acid composition of the mitochondrial genomes in Corvides. The *y*-axis represents the counts of corresponding amino acids utilized in various mitochondrial protein-coding genes across the Corvides species. The different colors represent the 20 different amino acids, as detailed in the figure legend.

Analysis of the 13 core mitochondrial proteins encoded by the mitogenomes of the Corvides revealed distinct compositional properties. Leucine was the most frequently used amino acid in 11 of the 13 proteins, highlighting its structural importance. In contrast, the MT-ATP8 and MT-ND6 proteins displayed a strong preference for Proline and Valine, respectively, indicating protein-specific functional requirements (Figure 7).

These 13 PCGs also exhibited conserved architectural features. The overall GC content of PCGs was relatively stable across the group, ranging from 41.46% (*Dicrurus megarhynchus*) to 46.49% (*Philentoma pyrhoptera*) (Table S6). Furthermore, we identified two non-canonical start codons, GTG (for *MT-CO1*, *MT-CO2*, and *MT-ND5*) and ATA (for *MT-ND1* and *MT-ND3*), which are common architectural features in the genes of vertebrate mitochondrial proteins (Table S7).

### 3.4. Phylogenetic Validation of Phylogenomic Signals

**Figure 8:**
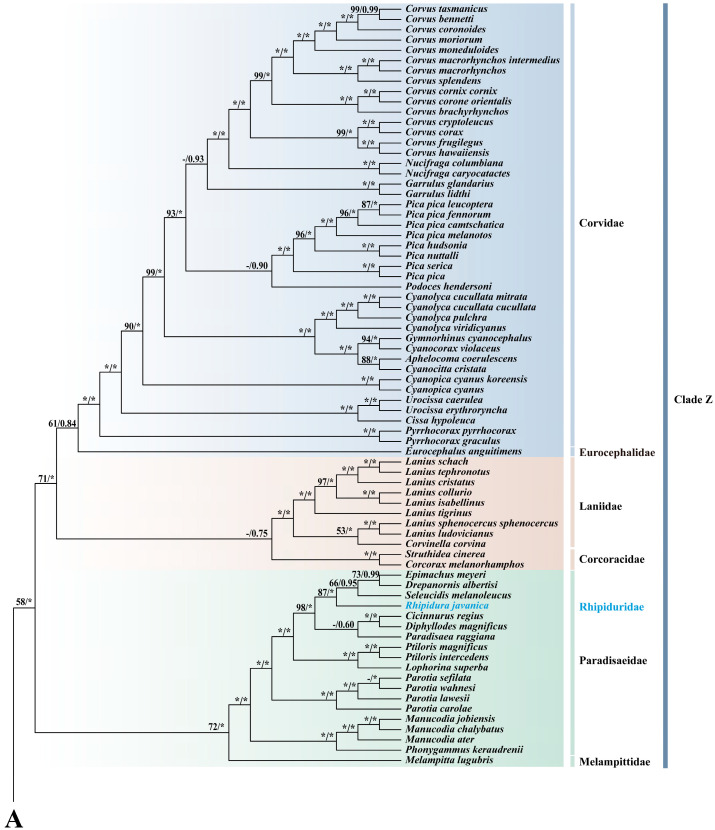

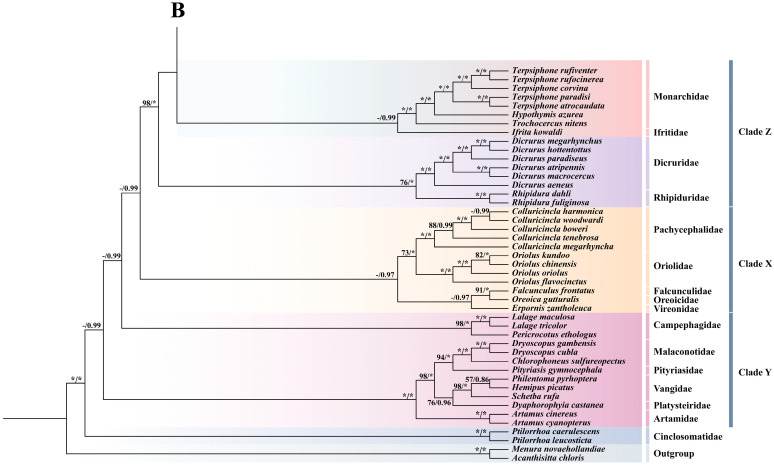
Phylogenetic tree of Corvides inferred from the concatenated nucleotide sequences of 13 mitochondrial protein-coding genes. (**A**) The upper part of the phylogenetic tree. (**B**) The lower part of the phylogenetic tree. The colored background blocks highlight different families and major clades (Clade X, Y, and Z). The tree illustrates the deep evolutionary relationships and branching order within the group. The Maximum Likelihood bootstrap support (BS) and Bayesian Inference posterior probability (PP) values for each node are indicated; the asterisk (*) indicates 100% BS or 1.00 PP, and the hyphen (-) indicates a support value below the threshold of 50, reflecting insufficient branch support or low topological confidence.

The robustness of our multi-layered approach was validated by assessing whether the intrinsic sequence properties of these PCGs were powerful enough to resolve the group’s deep phylogenetic history. Phylogenetic trees were reconstructed based on the concatenated nucleotide sequences of the 13 protein-coding genes, using both Maximum Likelihood (ML) and Bayesian Inference (BI) methods. The topologies from both analyses were largely congruent, revealing a well-resolved backbone for the group (Figure 8).

The result strongly supported the monophyly of Corvides. Nuclear data resolved the branching order (Orioloidea, (Malaconotoidea, Corvoidea)) [11,75], whereas our mitogenomic analyses recovered the distinct branching order (Corvoidea, (Orioloidea, Malaconotoidea)). The phylogenetic placements of ancient basal lineages within Corvides were resolved as follows: Ptilorrhoa (Cinclosomatidae) was strongly supported as the earliest-diverging clade of Corvides (BS = 100, PP = 1.00). Falcunculus and Oreoica formed a well-supported sister group (BS = 91, PP = 1.00). Ifrita (Ifritidae) was placed as the sister taxon to Monarchidae, with low ML bootstrap support (BS = 42) but high Bayesian posterior probability (PP = 0.99). Finally, both phylogenetic methods consistently placed Eurocephalus at the base of Corvidae, as the sister group to the clade comprising the remaining 46 sampled corvid taxa (BS = 61, PP = 0.84). The ability to resolve these deep nodes demonstrates that these PCGs retain strong phylogenetic signal at the deep phylogenetic level, consistent with their slow evolutionary rates and strong functional constraints.

## 4. Discussion

This study provides a comprehensive investigation into the evolutionary dynamics of mitochondrial PCGs and their protein products within the Corvides, one of the major avian radiations. By integrating multi-level analyses of sequence architecture, selective pressures, and three-dimensional structure, our work revealed how fundamental principles of molecular evolution shape the form and function of these essential biological components.

### 4.1. Functional Constraints Are Physically Manifested in Protein Structure

Our analysis provides compelling evidence that the 13 core mitochondrial proteins are governed by intense functional constraints, and we offer a physical basis for these constraints. The consistently low dN/dS ratios (all <0.162) quantify the strong purifying selection acting to preserve their amino acid sequences, underscoring their critical role in the oxidative phosphorylation pathway, where functional integrity is paramount for cellular energy homeostasis [76,77,78,79]. This finding aligns with previous studies across diverse vertebrate taxa, which universally highlight the conservative nature of the mitochondrial proteome [80,81]. Crucially, our study extends beyond these established sequence-level statistics by providing a physical interpretation of these constraints through 3D structural modeling. The stark contrast between the most conserved protein, MT-CO1, and a representative fast-evolving protein, MT-ND2, serves as a powerful case study. In MT-CO1, the clustering of highly conserved residues within the transmembrane helices that form the essential proton-pumping channel vividly illustrates how purifying selection acts to preserve the structural scaffold [82,83,84,85,86]. This conservation extends beyond secondary structure, ensuring the precise 3D arrangement of residues for proton transfer and the proper docking surfaces for other nuclear-encoded subunits. This reveals a dual functional constraint: maintaining both the protein’s intrinsic catalytic activity and its integration into the larger, co-evolved Complex IV assembly [87,88,89,90]. Conversely, the high variability on the surface and in the loop regions of MT-ND2 highlights areas of relaxed functional constraint. These regions are likely less critical for the protein core catalytic function and may be more involved in modulating interactions with the lipid bilayer or other subunits, thus tolerating a higher degree of amino acid substitution [91,92,93,94]. This pattern of a conserved core surrounded by variable loops is particularly evident in MT-ATP8. As the most rapidly evolving protein in our dataset, its evolution may be linked to its smaller size and role as an accessory subunit, where its evolution could be driven by co-evolutionary dynamics with nuclear-encoded ATP synthase components or a compensatory-feedback process [78,95,96,97].

### 4.2. Sequence Architecture Is Optimized for Efficient Protein Biosynthesis

Having established the functional constraints on the final protein products, we then investigated the underlying sequence architecture responsible for their biosynthesis. Our findings indicate that natural selection, rather than mutational pressure, is the principal architect of codon usage patterns in Corvides mitogenomes. This conclusion is supported by multiple lines of evidence, including ENC-GC3s plots and neutrality analyses. We propose that the significance of this finding lies not in the evolutionary mechanism itself, but in the optimization of protein biosynthesis. In a highly expressed system like the mitochondrion, selection for specific codon architectures that enhance the speed and accuracy of translation confers a significant fitness advantage by ensuring the correct folding and stability of the final protein products [98,99,100]. The significant preference for “optimal” codons that form perfect Watson–Crick pairs with their corresponding tRNA anticodons provides a clear mechanistic link supporting this hypothesis, directly enhancing translational speed and fidelity to minimize wasteful biosynthesis [101,102,103].

Notably, *MT-ND6* and *MT-ATP8* showed relatively higher dN/dS ratios, suggesting distinct evolutionary constraints compared with other mitochondrial PCGs. The codon usage pattern of *MT-ND6*, with a preference for G and T at the third codon position, differs from that of most other mitochondrial genes and may be related to its location on the mitochondrial light strand. This complementary nucleotide pattern suggests that strand-specific mutational biases, or possibly an antisense-strand evolutionary origin, may have contributed to its unique sequence composition. In contrast, although *MT-ATP8* also evolves relatively rapidly, its codon usage pattern is similar to those of other mitochondrial PCGs, indicating that its evolution may be shaped mainly by locus-specific selective constraints rather than strand-specific nucleotide biases.

However, the observation that overall codon usage bias is relatively weak (as indicated by ENC values) yet is shaped by strong selective forces presents an interesting paradox [104,105,106]. It suggests a dynamic equilibrium where selection for translational efficiency is constantly counteracted by the inherent mutational biases of the mitochondrial genome, resulting in a complex but functionally optimized sequence landscape.

### 4.3. Codon Aversion Motifs as Lineage-Specific Translational Signatures

Building on the analysis of codon usage, our identification of lineage-specific Codon Aversion Motifs (CAMs) introduces a novel layer of architectural information that can be interpreted as an extreme form of translational regulation. While codon usage bias describes general preferences, CAMs represent the complete aversion of specific codons within a given evolutionary lineage. The biological mechanisms driving CAMs are likely multifaceted, possibly involving co-evolution with the nuclear-encoded translational machinery, the loss or modification of specific tRNA genes, or selection against codons that are prone to translational errors [107,108]. Regardless of the underlying cause, our results demonstrate that CAMs are stable within clades and variable between them, making them a promising source of phylogenetic signal, particularly for resolving rapid radiations where traditional sequence data may be less informative.

### 4.4. Compositional Properties Reflect Structural and Evolutionary Conservation

The basic compositional properties of these components provide a stable background for these dynamic evolutionary processes. The high frequency of hydrophobic amino acids, such as Leucine, is consistent with the primary role of these proteins as integral components of the inner mitochondrial membrane, reflecting a fundamental structural requirement [109,110,111]. Furthermore, the overall stability of GC content and the conservation of non-canonical start codons across the group are characteristic of vertebrate mitogenomes [80,81]. This underlying conservation reinforces the idea that the entire system, from the genetic code to the final protein products, is a tightly co-evolved unit, providing a stable foundation upon which the functional constraints and selective pressures we observed can act.

### 4.5. Phylogenetic Reconstruction Validates the Multi-Layered Approach

The robustness of our multi-layered approach was validated by the ability of the intrinsic sequence properties of these PCGs to resolve the group’s deep phylogenetic history. The successful reconstruction of a well-supported phylogeny, which resolves deep nodes within Corvides with high confidence (e.g., Ptilorrhoa as the earliest-diverging clade, BS = 100, PP = 1.00), demonstrates that these PCGs retain strong phylogenetic signal at the deep level. Notably, the recovered branching order among the three major superfamilies (Corvoidea, (Orioloidea, Malaconotoidea)) differs from the nuclear consensus (Orioloidea, (Malaconotoidea, Corvoidea)) [11,75]. This mito-nuclear discordance is consistent with observations in other avian clades [112,113] and likely reflects the distinct evolutionary histories of mitochondrial and nuclear genomes. Importantly, this discordance does not undermine the phylogenetic value of these PCGs. Instead, it shows that the sequence properties we characterized—composition, codon usage, and selective pressure—reflect a genuine mitochondrial evolutionary history, different from but equally valid as the nuclear perspective. The fact that these PCGs can still resolve deep nodes, regardless of topology, confirms that they carry a clear evolutionary record and supports the foundation of our approach.

## 5. Conclusions

In conclusion, this study offers a detailed examination of the forces shaping the 13 PCGs and core mitochondrial proteins in the Corvides. By integrating analyses of 3D structure, functional constraint, and sequence-level architecture, we have revealed how these essential components of the cellular energy machinery are maintained and fine-tuned by evolution. Our work establishes a framework for future studies, demonstrating that the intrinsic properties of PCGs and their protein products reflect their structure, function, and evolutionary history.

## Figures and Tables

**Table 1:** Evolutionary rates of mitochondrial PCGs of Corvides species.

Gene	Length (bp)	PV (%)	π	dN	dS	dN/dS
*MT-ATP6*	681	58.44	0.17674	1.9759	72.2786	0.02733
*MT-ATP8*	165	69.70	0.1903	4.1706	25.7216	0.16214
*MT-CO1*	1563	40.91	0.12470	0.3132	40.2068	0.00779
*MT-CO2*	690	49.28	0.14739	1.1758	38.6019	0.03046
*MT-CO3*	783	47.89	0.13931	0.8595	44.2873	0.01941
*MT-CYB*	1140	50.35	0.14964	1.4031	48.7720	0.02877
*MT-ND1*	978	52.25	0.17481	1.2640	59.3141	0.02131
*MT-ND2*	1039	66.41	0.19699	2.8469	46.0301	0.06185
*MT-ND3*	348	55.46	0.16920	2.4047	41.6757	0.05770
*MT-ND4*	1383	55.17	0.16331	1.6308	61.3022	0.02660
*MT-ND4L*	294	59.52	0.17179	1.6050	51.7562	0.03101
*MT-ND5*	1833	58.92	0.17102	2.0708	58.0352	0.03568
*MT-ND6*	519	62.81	0.19059	3.0585	35.6864	0.08570

## Data Availability

The data presented in this study are openly available in the GenBank database under the accession numbers BK072017–BK072067. Detailed information regarding these sequences can also be found in Table S1 of the Supplementary Materials.

## Supplementary Materials

The following supporting information can be downloaded at: https://www.mdpi.com/article/10.3390/biology15141190/s1, Table S1: The SRA accession numbers of Corvides species; Table S2: The best Bayesian evolutionary models in the mitogenomic dataset; Table S3: The detailed ENC values of 13 mitochondrial genes among Corvides species; Table S4: The RSCU values of codons among Corvides species; Table S5: The overall over-represented codons and preferred codons in the mitochondrial genes among Corvides species; Table S6: General Characteristics of Corvides Mtgenomes; Table S7: The start and stop codons used in mitogenomes of Corvides species; Figure S1: Neutrality plots of mitochondrial genes among Corvides species.
