# Supplementary figures and images for: From Codons to Protein Structure: Evolutionary Constraints of Mitochondrial Proteins in Corvides
Source: Biology (Basel). 2026 Jul 19;15(14):1190. doi: 10.3390/biology15141190 (PMC13404721; doi:10.3390/biology15141190)

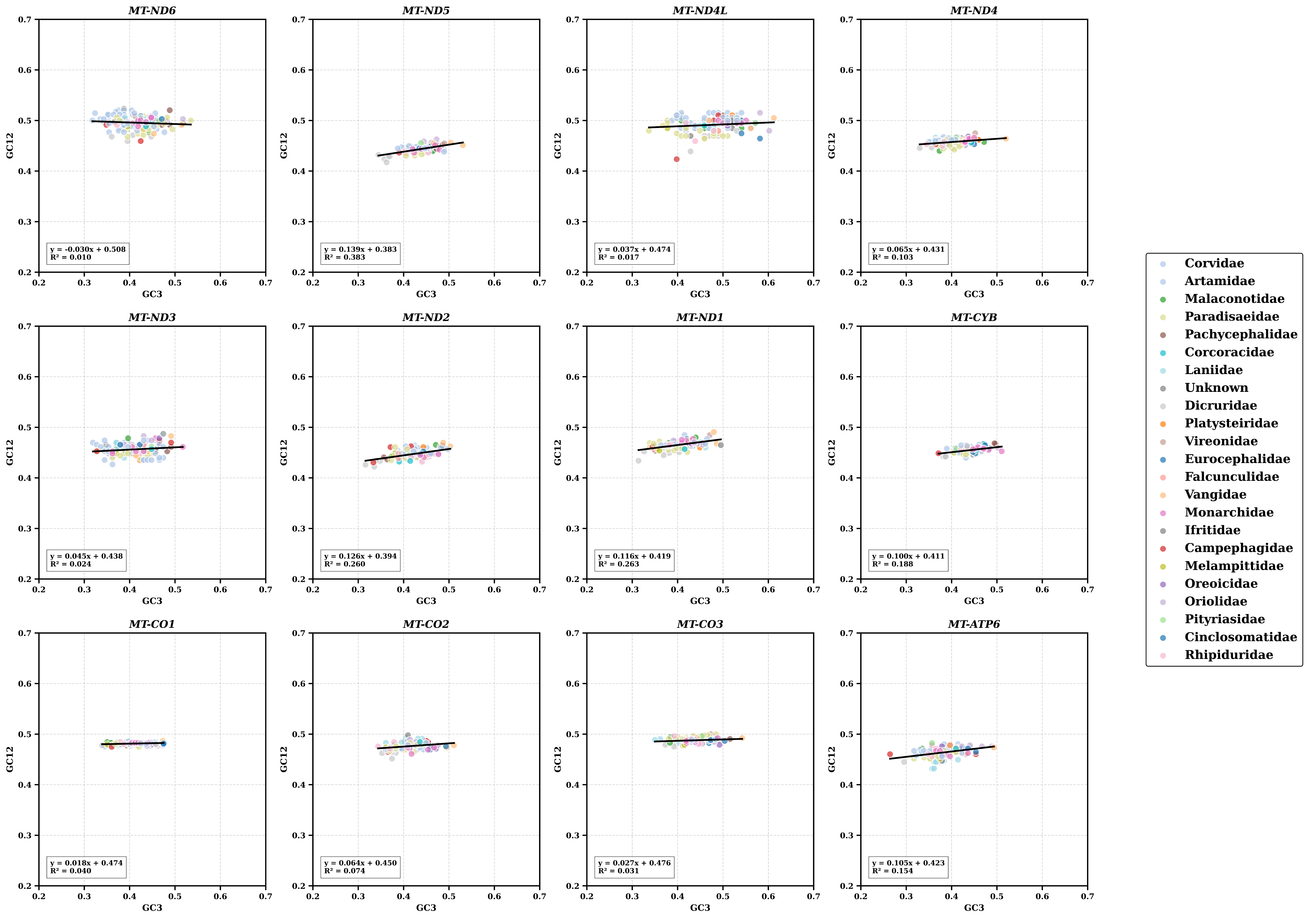

Supplement: Supplementary file 1 [file biology-15-01190-s001.zip › Figure S1.tif]
